# Low-Cost Additive Manufacturing Techniques Applied to the Design of Planar Microwave Circuits by Fused Deposition Modeling

**DOI:** 10.3390/polym12091946

**Published:** 2020-08-28

**Authors:** Héctor García-Martínez, Ernesto Ávila-Navarro, Germán Torregrosa-Penalva, Alberto Rodríguez-Martínez, Carolina Blanco-Angulo, Miguel A. de la Casa-Lillo

**Affiliations:** 1 Materials Science, Optical and Electronic Technology Department, Miguel Hernández University of Elche, 03202 Elche, Spain; eavila@umh.es (E.Á.-N.); cblanco@umh.es (C.B.-A.); 2 Communication Engineering Department, Miguel Hernández University of Elche, 03202 Elche, Spain; gtorregrosa@umh.es (G.T.-P.); arodriguez@umh.es (A.R.-M.); 3 Bioengineering Institute, Miguel Hernandez University of Elche, 03202 Elche, Spain; mcasa@umh.es

**Keywords:** high-frequency circuits, material characterization high-frequency, rapid prototyping, ultrasonic characterization, 3D printing

## Abstract

This work presents a study on the implementation and manufacturing of low-cost microwave electronic circuits, made with additive manufacturing techniques using fused deposition modeling (FDM) technology. First, the manufacturing process of substrates with different filaments, using various options offered by additive techniques in the manufacture of 3D printing parts, is described. The implemented substrates are structurally analyzed by ultrasound techniques to verify the correct metallization and fabrication of the substrate, and the characterization of the electrical properties in the microwave frequency range of each filament is performed. Finally, standard and novel microwave filters in microstrip and stripline technology are implemented, making use of the possibilities offered by additive techniques in the manufacturing process. The designed devices were manufactured and measured with good results, which demonstrates the possibility of using low-cost 3D printers in the design process of planar microwave circuits.

## 1. Introduction

Additive manufacturing techniques and 3D printers have evolved in recent years as an essential technology that provides different solutions in a wide variety of industries, such as construction, medicine, aerospace, foods and education [1,2,3,4,5,6,7]. Three-dimensional (3D) printing is obtaining a significant interest in overcoming the challenges associated with traditional manufacturing processes, such as micromachining, due to its ease of manufacture, low production costs, and minimal waste of materials [8]. Recently, although it is still in the initial phase, additive technology is also exploring the possibility of making electronic and electromagnetic devices, thanks to the emergence of promising new materials, along with the possibility of implementing complex structures and geometries. For example, in electronics, 3D printers are being used for the manufacture of printed sensors, conformal electronics, and stretchable electronics [9,10,11,12,13,14,15]. For microwave devices, additive technology is primarily focused on the design of 3D structures, such as waveguide devices, antennas, and sensors [16,17,18,19,20,21,22].

Other features that 3D printing can offer are the use of different material densities, the use of multilayer devices with multiple dielectric layers, each with the necessary height, the design of the dielectric substrate for a specific application and even the use of different materials [23]. Of all the technologies in additive manufacturing today, fused deposition modeling (FDM) technology is used in this work because it allows the utilization of very low-cost 3D printers and a wide variety of conventional plastic-based filaments and the combination of plastics with other materials (Nylon, ceramic, wood, metals, conductors, etc.) that can be of great interest in the design of microwave planar circuits. Since all commercial filaments available for this type of 3D printer are not intended to implement microwave devices, it is necessary to obtain the electrical parameters of each material (dielectric constant and loss tangent). Additionally, since FDM does not allow the printing of metallic materials and the conductive filaments currently available do not have high conductivity [24], for microwave circuits it is necessary to develop a technique for the metallization of 3D printing parts, using copper plates attached directly to the substrate, as it is done with conventional high-frequency substrates [25,26].

Additionally, due to the fabrication process of the circuit, which comprises, on the one hand, the fabrication of a substrate based on a plastic pseudo-thermofused layered structure, and for the other, the use of epoxy adhesives to glue the copper boards, it is necessary to check if the whole process results in a reliable structure. Errors in any of the fabrication stages, such as voids or bubbles in between the thermoplastic layers or in the substrate–adhesive-copper interfaces, excess of adhesive, or lack of homogeneity in the density of the material layers, would compromise both the expected performance and the structural integrity of the resulting circuit [27,28]. The structural analysis is conducted using ultrasonic nondestructive techniques, as it is fast, inexpensive, and very accurate, and can be used without damaging the materials for their future use. The circuits are evaluated using both time and frequency domain analysis techniques based on deconvolution [29] and spectroscopy [30,31,32,33,34] respectively, applied to C-scans of the circuits to reveal any defect or structural problem.

This study shows the possibility of using low-cost additive techniques in the design of dielectric substrates with different materials, for its use in the manufacturing of typical and complex microwave circuits. For this purpose, it is shown the electrical performance of different filaments and the structural performance of the whole manufacturing process, and finally, to validate the 3D printing technology, a proof of concept is made, implementing simple microwave planar circuits in different microwave technologies. Following this manufacturing methodology, it is possible to implement more complex and specific structures of microwave circuits such as multilayer filters where the coupling factor of the different filter sections can be designed by properly configuring the 3D printer [35], obtaining higher bandwidths and increasing the rejection band in the undesired frequencies, or the design of waveguide filters by means of periodic structures where the additive techniques allow the design of the waveguide sections to obtain a higher rejection bandwidth [36].

This work is organized as follows: Section 2 describes the different materials used in this study, the characteristics of the printer and the manufacturing process of the microwave substrates; Section 3 presents the electrical characterization of any 3D print filament, the structural analysis of the different circuits and a proof of concept of the process of designing, manufacturing and measuring different stepped impedance filters in different microwave technologies using as a novelty the design of the dielectric properties; finally, the conclusions of this work is shown in Section 4.

## 2. Materials and Methods

### 2.1. Materials

In this work, various 1.75 mm diameter standard filaments were analyzed, seeking to obtain different electrical properties for the design of microwave circuits. The materials used were: PolyLactic Acid (PLA) filament from German RepRap Gmh, which is a polymer consisting of lactic acid molecules; Acrylonitrile Butadiene Styrene (ABS) filament from Fillamentum, which is an amorphous thermoplastic material very resistant to impact that is mainly employed for the development of industrial and automotive parts; Iglidur I180-PF (Tribo) filament from Igus, which is a friction resistant material up to 50 times more resistant than other materials such as ABS or PLA, since it offers a great response to wear degradation; Acrylonitrile Styrene Acrylate (ASA) filament from Fillamentum, which is a thermoplastic filament similar to ABS that combines mechanical robustness, resistance to ultraviolet rays and water; PLA Stainless Steel filament from Protopasta, which is made of a PLA polymer and a grinded filament from pulverized stainless steel and offers an appearance and density more similar to stainless steel; Laybrick filament from CC-PRODUCTS, which is a filament made of sandstone and a bonding polymer that recreates a surface finish similar to ceramic or stone objects; Nylon 230 filament from Taulman, which is a synthetic polymer from the group of polyamides; LayWoo-D3 filament from CC-PRODUCTS, which is a filament manufactured from wood fibers (40%), PLA (50%) and a bonding polymer, and recreates a surface finish and texture similar to wood; and finally, Smartfil EP filament from Fillamentum, which is a material composed of PLA (70%) and Calcium Carbonate (30%), and presents a surface finish similar to limestone.

### 2.2. D Printer

**Figure 1:**
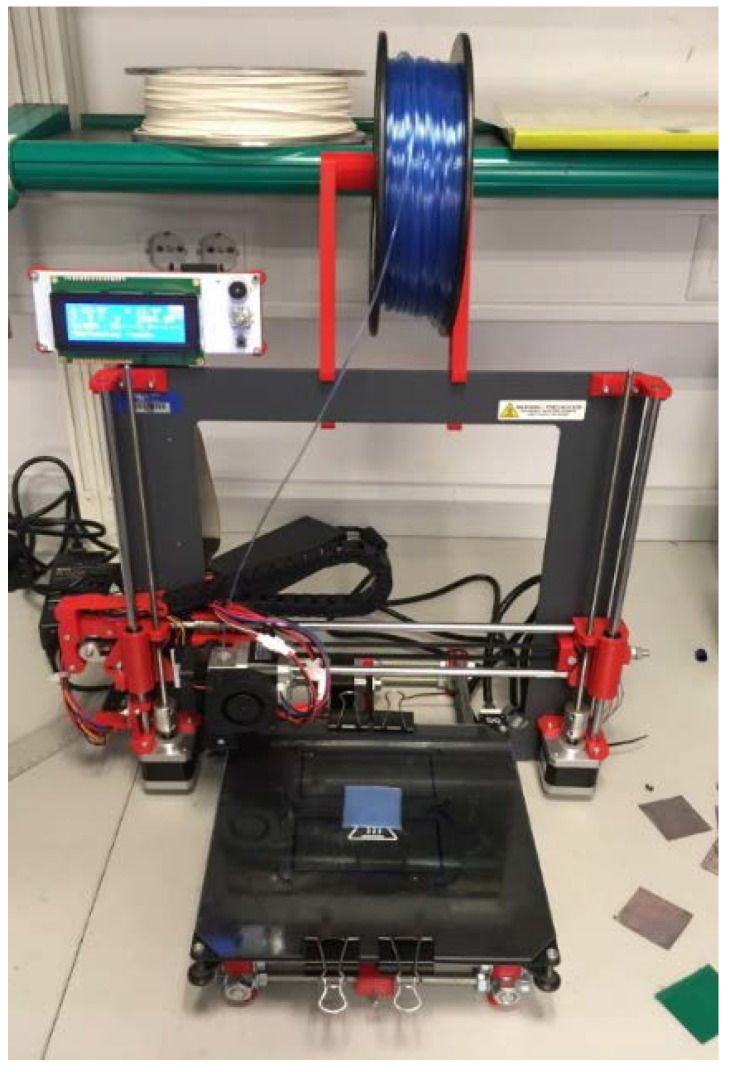
Prusa i3 BQ Hephestos 3D Printer used in this work. (Photo from BQ Hephestos).

For the use of the different filaments presented in the previous section, a low-cost 3D printer Prusa i3 Hephestos (from BQ) model was used, as shown in Figure 1. This type of 3D printer allows printing a maximum volume of 225 × 210 × 280 mm^3^ with 60 µm of horizontal and vertical resolutions [37].

This 3D printer uses the FDM technology, which allows printing a wide variety of materials and to employ many adjustable options that affect the final properties of the 3D printed objects (substrate laminates for microwave circuits implementation in our case) including: extruder speed, extrusion temperature, thickness, height of the initial layer, as well as the filling pattern density. Three-dimensional (3D) printer Cura software from Ultimaker [38] was used to adjust the different 3D printing control parameters for each material employed, as it is given in Table 1.

### 2.3. Manufacturing Process

**Figure 2:**
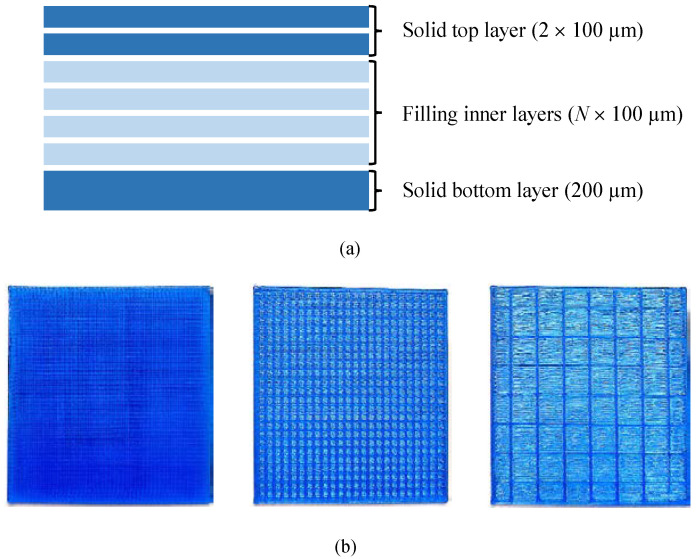
(**a**) Structure of the printed layers of the substrates. (**b**) Linear printing pattern of 3D material with different filling densities, 100%, 50% and 15%.

The different sheets that form the 3D printed object (microwave circuit substrates) can be classified into outer layers and inner layers, as shown in Figure 2a. The thickness of the outer layers especially affects the mechanical stiffness of the substrate. Therefore, the outer layers should be solid with a filling pattern density percentage of 100%. The pattern adopted in the design of these layers is usually rectilinear, to avoid porosity and reduce the roughness of the surfaces, while in the inner layers any pattern and filling density can be used. The bottom sheet has a thickness of 200 µm with a filling density of 100%. Different N sheets of internal 100 µm thick layers are stacked until the desired height is produced, where different filling density percentages, from 100% to 15%, can be considered with the aim of modifying selectively the electrical (and mechanical) characteristics of the substrate, as can be seen in Figure 2b. The upper part of the substrate consists of two solid 100 µm thick sheets (also with a filling density of 100%). After analyzing different filling pattern alternatives, the rectilinear pattern was used for all layers because it provides the most reliable pattern for substrate manufacturing, and allows carrying out the printing process more easily. Filling densities lower than 15% produce substrate laminates that lack the required rigidity.

**Figure 3:**
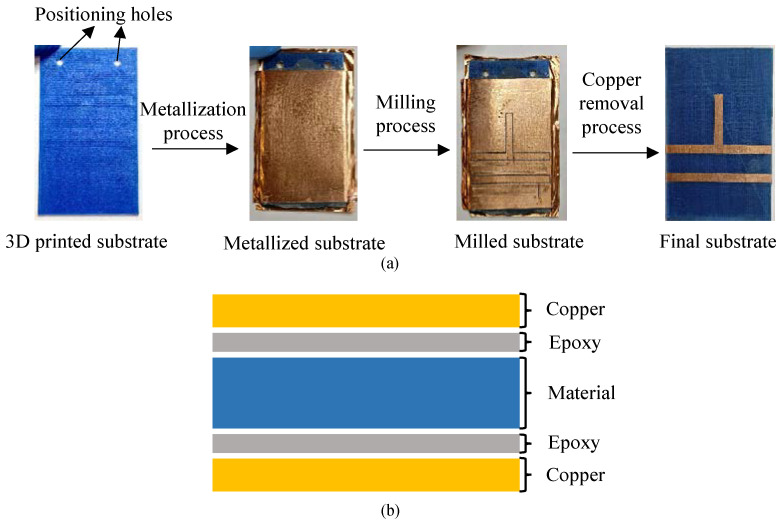
(**a**) Printed circuit board manufacturing process. (**b**) Printed circuit structure of the different materials.

Once the 3D printing substrate was manufactured, it is metalized by gluing two 35 μm copper sheets from Basic Copper on both sides, using a thin layer of nonconductive 2216 B/A epoxy glue GRAY from 3 M applied manually with a brush. For a correct bonding, the pressure of 15.3 kgf/cm^2^ is applied by means of a hydraulic press to the two copper sheets for one hour, with a room temperature of 23 °C and a humidity of 30%. Once the epoxy glue solidifies, the substrate is ready to be used. A Protomat S42 from LPKF numerical control milling machine is used in the fabrication of the microwave circuits. Figure 3a describes the manufacturing process of the printed circuit and Figure 3b shows the different layers of the implemented substrate.

## 3. Results and Discussion

Once the different filaments were presented and the manufacturing method in the 3D printer explained, this section describes the electrical characterization of the different materials, the structural analysis of the manufacturing process and the joining of the different copper sheets, and finally, a proof of concept is made by designing and manufacturing simple planar microwave circuits that demonstrate the feasibility of using this technology.

### 3.1. Electrical Characterization at Microwave Frequencies

**Figure 4:**
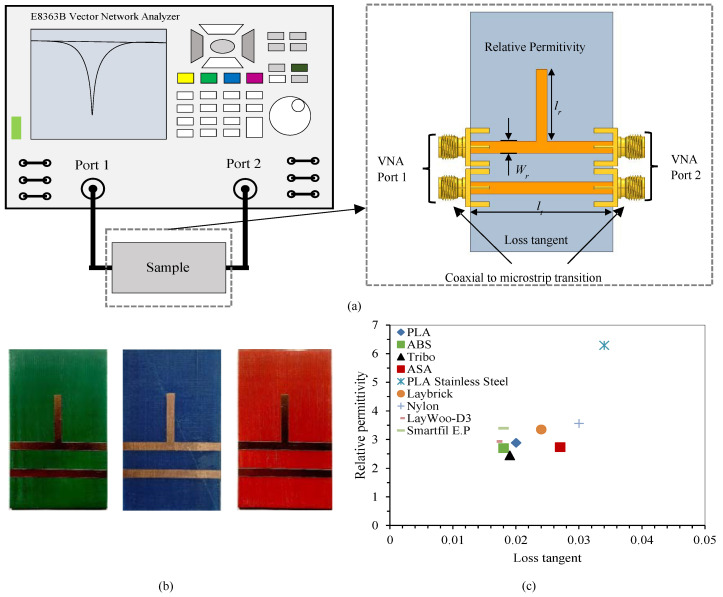
(**a**) Setup for the dielectric permittivity and loss tangent measurements. (**b**) Resonator and transmission line on different materials: Acrylonitrile Butadiene Styrene (ABS), PolyLactic Acid (PLA) and Acrylonitrile Styrene Acrylate (ASA). (**c**) Electrical characteristics calculated for different substrates.

Low-cost 3D printing filaments are not specifically intended for the design of microwave circuits, therefore printed materials require the characterization of their electrical properties, in terms of relative permittivity and loss tangent. The electrical characteristics of the substrate depend on the material used, the height of the substrate layers and the configuration of the printer, mainly the density of the layers. For the electrical characterization of materials at high frequencies, different techniques can be used, including cavity resonators and coaxial probes [39,40]. In this work, we use a quarter wavelength resonator based on an open-ended transmission line to calculate the relative permittivity, and a transmission line to determine the losses of the material, as shown in Figure 4a because it takes into account the entire substrate manufacturing process and provides reasonably accurate results in the microwave range. Both the resonator and the transmission line were designed using the EMPRO commercial electromagnetic simulator and the Advanced Design System (ADS) circuit simulation software (both from Keysight Technologies, Santa Rosa, CA, USA). The length of the resonator (*l_r_* = 19.2 mm) was selected to provide a resonant frequency around 2.5 GHz, for a dielectric height of 1.6 mm and an estimated substrate dielectric permittivity around 3.0, while the transmission line length (*l_t_* = 50 mm) is designed to have significant transmission losses to allow the extraction of the material loss tangent. In both cases, the width of the microstrip lines (*W_r_* = 4 mm) was selected to present a characteristic impedance of 50 Ω so that the mismatch losses are minimal. Several circuits made with different filaments for 3D-printing are shown in Figure 4b. Once all the testing circuits with the different substrate materials were manufactured, the response of both circuits, the resonator and the transmission line, is measured using the vector network analyzer (VNA) E8363B (from Keysight Technologies, Santa Rosa, California, USA), as shown in Figure 4a. For each material, the dielectric permittivity and the loss tangent are optimized with ADS and EMPRo simulators so that simulations fit the measured responses. Figure 4c shows the electrical characteristics of all the materials used.

**Figure 5:**
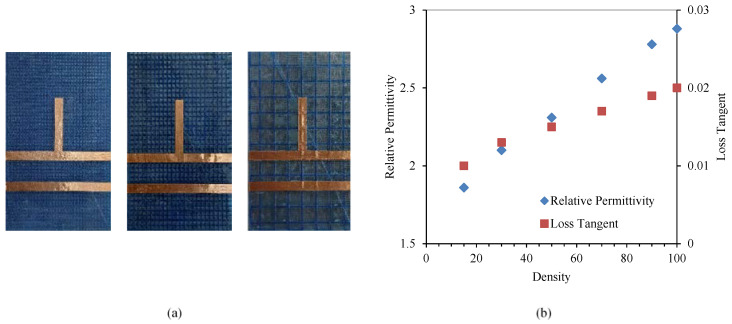
(**a**) Resonator and transmission line for PLA with 70%, 50% and 15% densities. (**b**) Electrical characteristics for different filling densities of the PLA substrate.

Additionally, the electrical performance of different PLA substrates was carried out by varying the filling percentage of the inner layers, as shown in Figure 5a. The total height for each substrate is 1.6 mm while maintaining in all cases a filling density pattern of 100% for the outer layers. By modifying the filling percentage of the inner layers, it is possible to vary the relative permittivity of the substrate as well as its loss tangent [41]. Figure 5b shows the electrical characteristics of the PLA for different filling densities. As expected, lower filling density leads to a reduced relative permittivity and loss tangent of the material.

From the 3D printing filament previously characterized, PLA was finally chosen to perform a structural analysis of the manufacturing process and a proof of concept with different substrates geometries, to design and implement various microwave circuits because this material is relatively inexpensive, has similar electrical characteristics to FR4 (which is a low-cost material widely used in microwave circuits) and can result in the implementation of very economical microwave circuits. In addition, PLA is a rigid material, easy to manufacture in any low-cost printer (no heated bed required), has the highest printing speed of all the filaments used, and is a material that any small research laboratory can easily dispose of.

### 3.2. Structural Analysis

The structural analysis of the manufacturing process was conducted using simple pulse-echo ultrasonic nondestructive testing techniques. The circuits were scanned in an immersion basin in distilled water, using a 5 MHz focused transducer from OLYMPUS as pulse-echo transducer and 5 MHz pulses as excitation signals. For each circuit, the XYZ scanner performed two C-scans (2D scans along all the surface), one from each surface, top and bottom, taking A-scans (single measurement at a specific point on the surface of the circuit) every 200 μm. The pulser/receiver used as generator and acquisition equipment was an SE-TX06-00 from KTU electronics with a sampling rate of 100 MHz.

**Figure 6:**
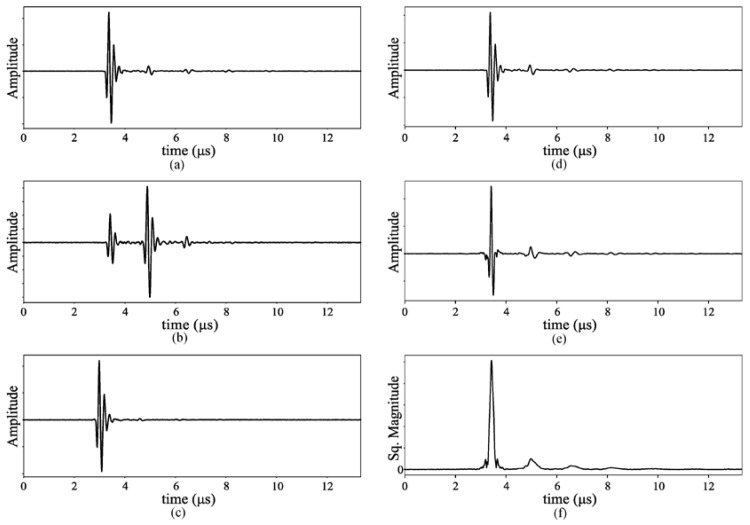
Examples of A-scans for different interfaces: (**a**) copper–copper, (**b**) PLA-copper and (**c**) copper–PLA. (**d**) Unprocessed A-scan, (**e**) compressed A-scan after Wiener filter and (**f**) its envelope.

The resulting A-scans (Figure 6a for copper–copper, Figure 6b for PLA-copper and Figure 6c for copper–PLA interfaces) were processed using simple time domain and frequency domain techniques. In time domain, magnitude C-scans where produced at different depths in the circuits by showing the magnitude at successive times of processed A-scans, which reveal the inner structure of the circuits, assuming that significant differences in magnitude and new echoes appear where the acoustic impedance changes, which happen especially with voids, bubbles and interfaces between materials, that is, at PLA-adhesive-copper interfaces. Because of possible bending of the circuits due to the manufacturing and curing process, which cause a misalignment in the echoes even if they come from the same structure, C-scans were first aligned to the bottom surface using a single A-scan as reference for the correlation and shifting, as described in [29], which includes a two-step deconvolution that also provides time-of-flight maps from the top and bottom surfaces. Resulting time-of-flight difference C-scans or B-scans (scans along one line in the surface), can be useful also to obtain the final dimensions of the structure with very high resolution (±1 µm), which is of interest to analyze the curing process of the different 3D printed substrates.

**Figure 7:**
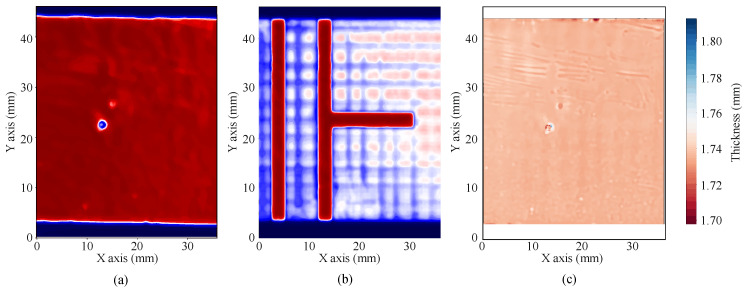
Examples of C-scan for (**a**) bottom surface, (**b**) top surface and (**c**) thickness map.

After alignment, to reduce the grain noise and enhance the resolution, A-scans are compressed using a reference A-scan as Wiener filter, obtained from a copper reference reflector. Then, the envelope of each compressed A-scan is calculated as the absolute value of its Hilbert Transform, whose magnitude is used to produce the final C-scans for both sides of the circuits. Figure 6 shows examples of the different stages of the process, with the original A-scan (Figure 6d), the compressed signal (Figure 6e) and the resulting envelope magnitude (Figure 6f). Figure 7 shows examples of processed magnitude C-scans from the top (Figure 7a) and bottom (Figure 7b) surfaces, as well as a thickness map (Figure 7c) obtained using deconvolution. These images are a clear example of the information that this simple analysis can provide to the circuit designer, that includes the thickness map, whose variations can affect the resulting dielectric properties of the substrate, and the state of the surfaces, including small scratches in the copper (below 1 μm) or adhesive excesses.

**Figure 8:**
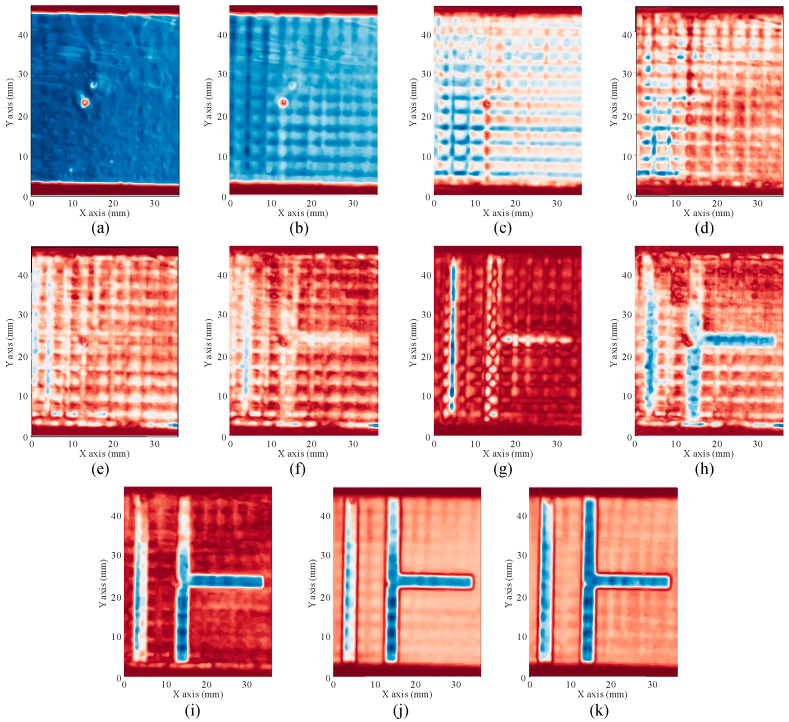
From left to right and top to bottom, successive layers inside the circuit in steps of 150 µm approximately.

Additionally, C-scans of the inner structure can be analyzed, providing information about the immediate area below the copper sheets and deeper layers, as well as filament misalignment, bubbles or voids in between filaments, changes in the density of some filaments, which could be caused by changes in the curing or extrusion temperatures, etc. Next figures (Figure 8) show examples of C-scans acquired at different depths (every 150 µm approx.), where the latticed structure of the different layers of the substrate can be seen, as well as some irregularities in the adhesion of the top surface (see the upper part of Figure 8h,i).

Regarding the frequency analysis, ultrasonic resonance imaging C-scans of the circuits were obtained [30,31,32,33,34]. Resonant spectrums were calculated as the transfer function between the A-scans obtained from the circuit and the aforementioned reference A-scan.

**Figure 9:**
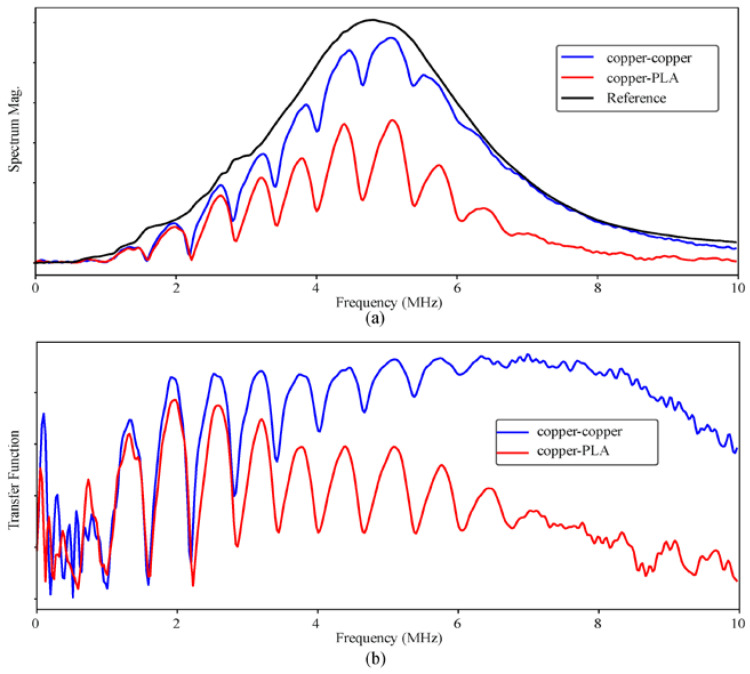
(**a**) Examples of spectrums of A-scans at different interfaces compared with the spectrum of the reference (copper) A-scan. (**b**) Examples of resulting resonant transfer functions.

Echoes from voids, bubbles and any other significant change in the acoustic impedance will resonate in such structures, producing local maxima in the transfer function spectrum (Figure 9a,b for the spectrums and resonant spectrums respectively), which can be explored in successive C-scans obtained along the frequency axis of the transfer function spectrum of all the A-scans. Combined with the magnitude C-scans, this analysis can be used to discriminate false defects or inhomogeneities, as it is less sensitive to the misalignment of the surfaces.

**Figure 10:**
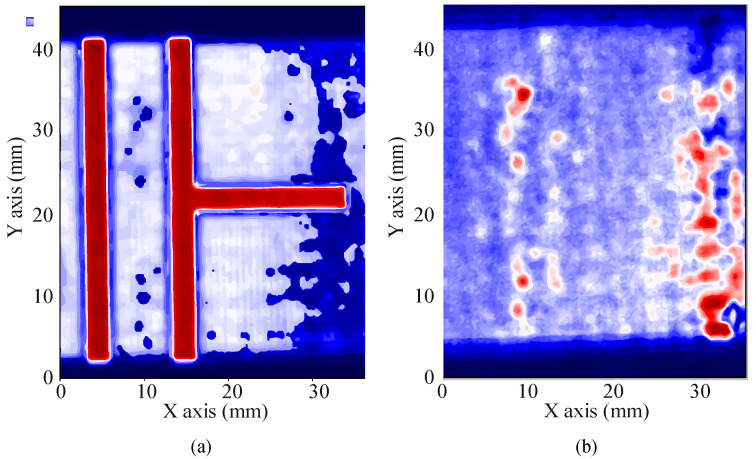
Examples of adhesion errors seen using resonant spectroscopy (**a**) and time analysis (**b**).

Figure 10 shows an example of a C-scan obtained using resonant spectroscopy (Figure 10a) and time analysis (Figure 10b) for the same circuit, in which the errors in the adhesion of the bottom layer are clearly visible, which were not noticed in the visual analysis.

The aforementioned methods, despite being quite simple, are very reliable and can be used without any experience in NDT evaluation by the circuit designers, therefore providing a valuable tool in the design stages at the laboratory. Additionally, as most of the specimens did not show relevant errors in the adhesion nor in the different materials’ structure, it proves that the method used for the design and assembly of the circuits is consistent, although a more detailed study of the proposed inspection methods, especially spectroscopy imaging, should be conducted in order to establish the relation between the resonant frequencies and the depth of the reflections.

### 3.3. Microwave Circuits Proof of Concept

To provide a proof of concept of the results obtained in the previous sections, stepped impedance filters are designed and manufactured in microstrip and stripline technologies because they are relatively simple to manufacture, are widely used in microwave systems, and allow easy comparison with traditional technologies. In particular, four fifth-order maximally flat low-pass stepped impedance filters centered at 2.0 GHz were designed and fabricated [41]. Two of the designed filters employ PLA substrate with a conventional constant density filling pattern (homogeneous substrate), while the other two are fabricated onto a PLA substrate specifically designed (for the implemented microwave circuits) by changing density filling pattern regions, which allows enhancing the filter performances. In all cases, a geometrical linewidth limit of 0.75 mm for the implementation of the filter high impedance sections was chosen, while for the low impedance sections the limit considered was 10.0 mm. Each series inductance *L* in the low-pass lumped element filter prototype is synthesized using a high characteristic impedance *Z_high_* line section whose length is determined by:

(1) $$l_{L}=\frac{LZ_{0}}{\beta Z_{high}}$$

while each parallel capacitance *C* in the low-pass lumped element filter prototype is synthesized using a low characteristic impedance *Z_low_* line section whose length is calculated using:

(2) $$l_{C}=\frac{CZ_{low}}{\beta Z_{0}}$$

where *Z*_0_ = 50 Ω is the reference impedance and *β =* 2π*/λ* is the phase constant at the design frequency, being *λ* the wavelength at the design frequency. Parameters *β*, *Z_high_* and *Z_low_* are dependent on the substrate dielectric permittivity.

#### 3.3.1. Microstrip Stepped Impedance Filters

A conventional microstrip low-pass filter design is implemented using a 1.6 mm high homogeneous substrate with a constant density filling pattern of 100%. As shown in Figure 9 for this material *ε_r_* = 2.88 and tan *δ* = 0.02. For this particular substrate, characteristic, *Z_high_* = 114.4 Ω and *Z_low_* = 26.5 Ω in microstrip technology, while for a 50 Ω characteristic impedance transmission line the width is 4.1 mm. Table 2 shows the length of the different microstrip filter sections.

**Figure 11:**
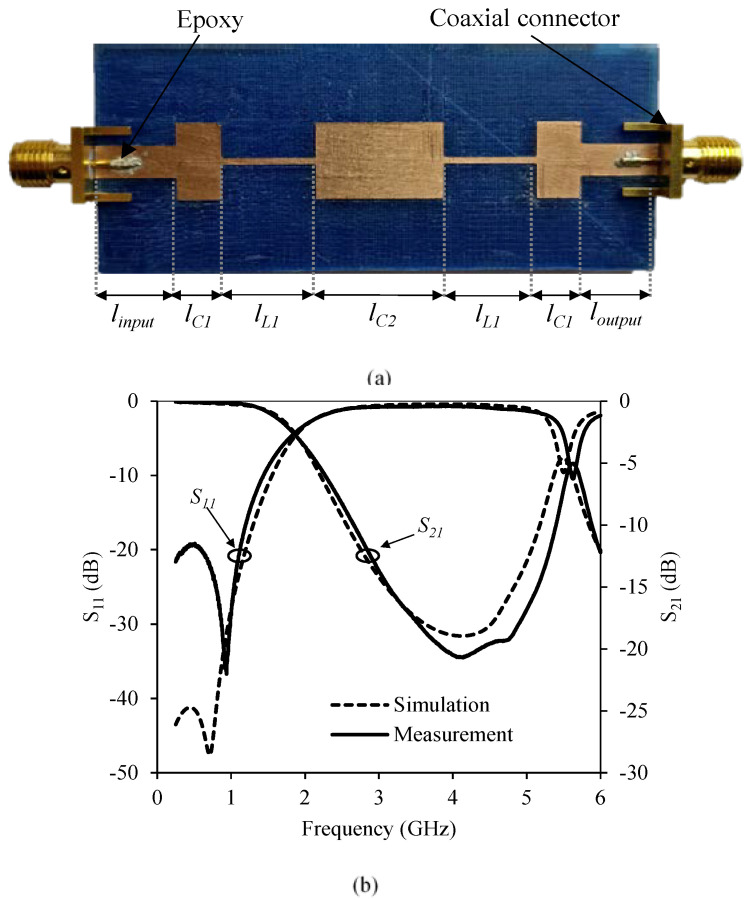
(**a**) Stepped impedance filter manufactured with 100% density. (**b**) Measured and simulated response of the stepped impedance filter with a 100% density substrate.

Figure 11a shows a photograph of the manufactured microstrip filter prototype including the SMA connectors. These connectors were attached using a silver conductive epoxy to improve the electrical connection and reduce losses. Connectors cannot be soldered because the temperature required exceeds the fusion temperature of PLA. Figure 11b shows a comparison between measured and simulated S-parameters. Both sets of curves are in very good agreement.

**Figure 12:**
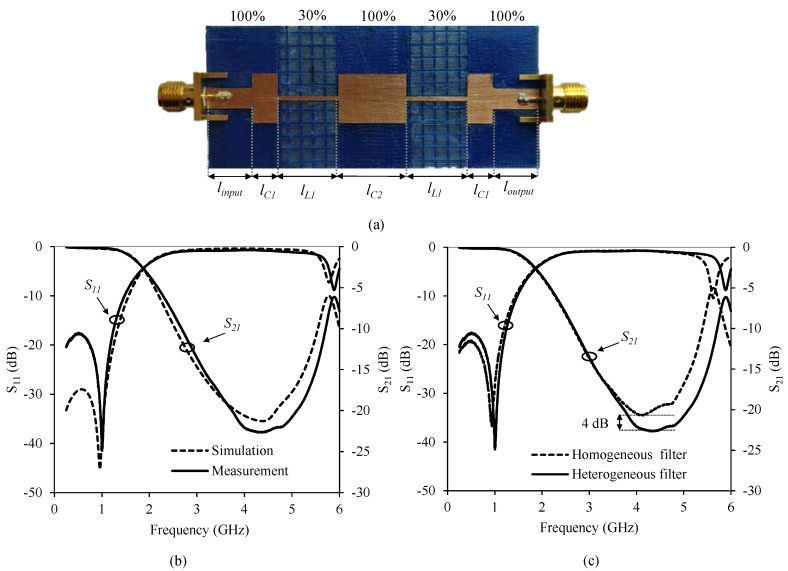
(**a**) Stepped impedance filter manufactured with different densities. (**b**) Measured and simulated response of the stepped impedance filter with different densities. (**c**) Comparison measurements of the different filters.

The second prototype implemented consists of a novel microstrip low-pass filter design using a 1.6 mm high heterogeneous substrate with different filling density patterns. As shown in Figure 12, a density filling pattern of 100% (with *ε_r_* = 2.88 and tan *δ* = 0.02) for *Z_low_* and a density filling pattern of 30% (*ε_r_* = 2.1 tan *δ* = 0.013) for *Z_high_* was used. For this particular substrate characteristics *Z_high_* = 129.1 Ω and *Z_low_* = 26.5 Ω in microstrip technology, providing a 14 Ohm increase for *Z_high_* compared with the previous filter. The width of a 50 Ω characteristic impedance transmission line is 4.1 mm. Table 3 shows the length of the different microstrip filter sections.

Figure 12a shows a photograph of the manufactured microstrip filter prototype including the SMA connectors. Figure 12b shows a comparison between measured and simulated S-parameters. Both sets of curves are in very good agreement. Figure 12c shows a comparison of the two microstrip filters, whereby using a heterogeneous substrate, an increase of 4 dB in the rejection band is achieved due to the use of a higher *Z_high_*.

#### 3.3.2. Stripline Stepped Impedance Filters

**Figure 13:**
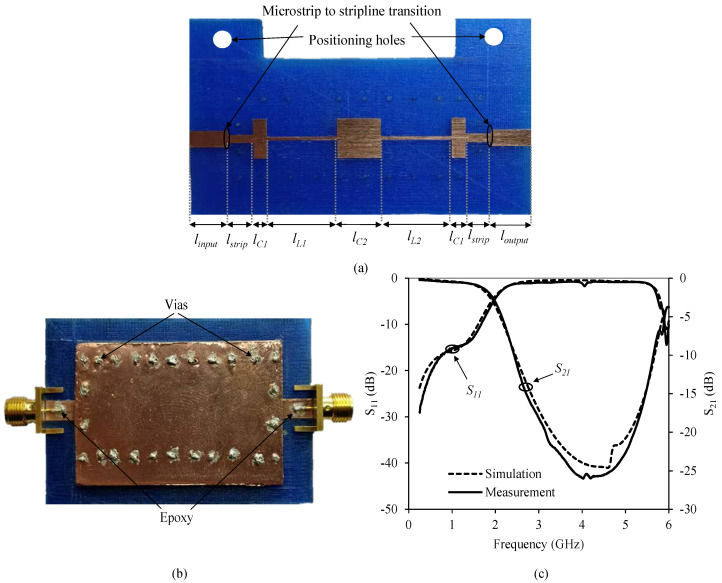
(**a**) Stripline stepped impedance filter with 100% density before completing its implementation. (**b**) Photograph of the fabricated filter. (**c**) Measured and simulated response of the stripline stepped impedance filter with a 100% density substrate.

Another feature of using additive 3D printing techniques is the possibility of easily making microwave devices in stripline or multilayer technology. The manufacturing process is similar to the previous one, but in this case, the substrate has to be grown again after engraving the circuit in the milling machine, so the correct positioning of the substrate both, in the milling machine and in the 3D printer, is critical. This process was carried out by adding positioning holes of about 3.0 mm diameter in the substrate piece. The first filter implemented aims to demonstrate the viability of using this type of technology by means of the design of a conventional low-pass filter in stripline technology, using a homogeneous substrate of 3.2 mm height with a filling density pattern of 100%, as it is shown in Figure 13a. For this type of material *ε_r_* = 2.88 y tan *δ* = 0.02, so for the designed substrate in stripline technology *Z_low_* = 7.92 Ω and *Z_high_* = 57.1 Ω, while the width of the 50 Ω transmission line is 3.86 mm. Table 4 shows the lengths of the different sections of the stripline filter.

Figure 13b shows a picture of the manufactured stripline filter prototype including the SMA connectors, and the metal vias. These connectors and vias were attached using a silver conductive epoxy to improve the electrical connection and reduce losses. Figure 13c shows a comparison between measured and simulated S-parameters, with a good agreement.

**Figure 14:**
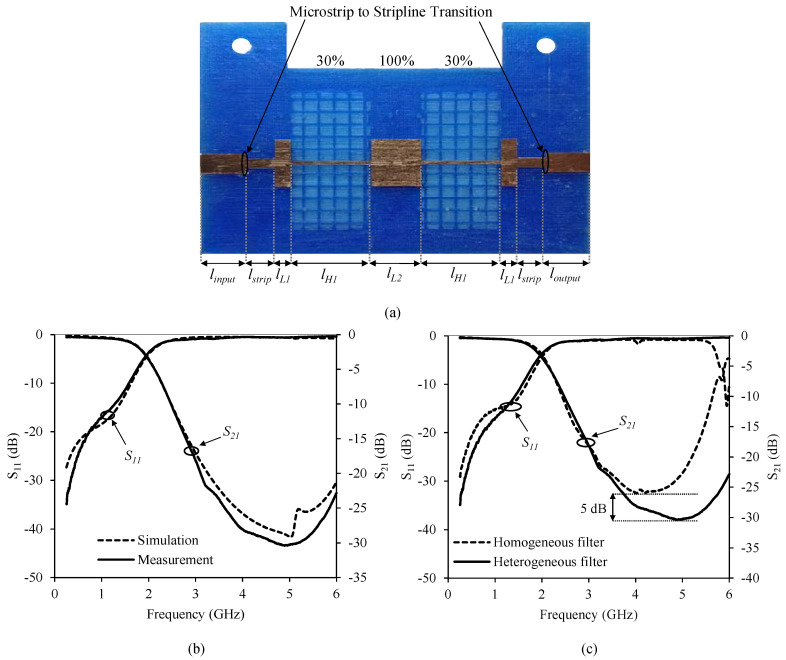
(**a**) Stepped impedance filter manufactured with different densities before its implementation is completed. (**b**) Measured and simulated response of the stepped impedance filter with different densities. (**c**) Comparison measurements of the different filters.

The second stripline prototype implemented consists of a novel low-pass filter design using a 3.2 mm high heterogeneous substrate with different filling density patterns. As shown in Figure 14a a density filling pattern of 100% (*ε_r_* = 2.88, tan *δ* = 0.02) for *Z_low_* and a density filling pattern of 30% (*ε_r_* = 2.1, tan *δ* = 0.013) for *Z_high_* was used. For this particular substrate in stripline technology, *Z_high_* = 65.1 Ω and *Z_low_* = 7.92 Ω, providing an 8 ohm *Z_high_* increase. The width of a 50 Ω transmission line is 2.5 mm. Table 5 shows the length of the different stripline filter sections.

Figure 14b shows a picture of the manufactured stripline filter prototype including the SMA connectors. Figure 14c shows a comparison between measured and simulated S-parameters. Both sets of curves are in very good agreement. Figure 14c shows a comparison of the two stripline filters, whereby using a heterogeneous substrate an increase of 5 dB in the rejection band is achieved.

According to the results presented in this article, FDM technology shows a high capacity of substrate configuration and good electrical characteristics to obtain microwave electronic circuits. These properties offered by 3D printing should be considered and exploited in future research work in order to obtain more complex microwave devices. The work presented includes a preliminary study on the characterization of materials for microwave applications with the main objective of developing high-frequency circuits with improved performance. The low cost, the different electrical properties that can be achieved, and the different configurations of the substrates, make 3D printing an interesting subject of research to make microwave devices faster, in a straightforward way and with better characteristics.

## 4. Conclusions

In this work, an incipient technology such as 3D printing was validated for the realization of simple and complex low-cost microwave circuits. The manufacturing process of the substrates, the structural analysis by ultrasound and the electrical characterization of different standard filaments was described, obtaining different relative permittivity and allowing to design the relative permittivity of a material by varying the filling density pattern and the total height of the substrate. The ultrasonic structural analysis has shown the reliability of the manufacturing process. Finally, to check the different possibilities offered by the additive manufacturing process presented, different simple and complex stepped impedance filters were implemented in microstrip and stripline technology. Good results were obtained in both technologies, with better performances when additive possibilities, such as different substrate densities are used, so it can be concluded that additive manufacturing techniques offer wide possibilities in the design of planar microwave circuits. The most interesting ones are the design of the substrate characteristics according to the needs of the application and the possibility of realizing complex structures to increase the performance of the microwave circuits.

## Figures and Tables

**Table 1:** Printing parameters of the used materials in the BQ Hephestos printer.

3D Printing Settings			
Material	Extruder Temperature (°C)	Bed Temperature (°C)	Extruder Speed (mm/s)
PLA	217	48	40
ABS	242	95	15
Tribo	256	100	20
ASA	255	100	20
PLA Stainless Steel	217	48	25
Laybrick	200	45	20
Nylon	237	50	15
LayWoo-D3	220	45	20
Smartfil EP	202	40	30

**Table 2:** Microstrip homogenous filter section length.

Parameter	Section Length (mm)
*l_input_*, *l_output_*	10.0
*l_C1_*	4.63
*l_L1_*	11.77
*l_C2_*	14.67

**Table 3:** Microstrip heterogeneous filter section length.

Parameter	Section Length (mm)
*l_input_*, *l_output_*	10.0
*l_C1_*	4.63
*l_L1_*	11.66
*l_C2_*	14.67

**Table 4:** Stripline homogenous filter section length.

Parameter	Section Length (mm)
*l_input_*, *l_output_*	10.0
*l_strip_*	5.0
*l_C1_*	4.63
*l_L1_*	10.78
*l_C2_*	14.67

**Table 5:** Stripline heterogeneous filter section length.

Parameter	Section Length (mm)
*l_input_*, *l_output_*	10.0
*l_strip_*	5.0
*l_C1_*	4.63
*l_L1_*	10.78
*l_C2_*	14.67
